# Modern Dentistry and the Tuberculosis Problem

**Published:** 1910-09-15

**Authors:** S. Adolphus Knopf

**Affiliations:** Professor of Phthisiotherapy at the New York Post-Graduate Medical School and Hospital. New York


### MODERN DENTISTRY AND THE TUBERCULOSIS PROBLEM.

S. ADOLPHUS KNOPF, M.D.

Professor of Phthisiotherapy at the New York Post-Graduate Medical School and Hospital.
NEW YORK

The latest and the most glorious development of American dental science is dental hygiene. It is this subject which brings me to my topic, for dental hygiene means prevention and preservation, and these bear the closest relation to the prevention of tuberculosis.

One of the earliest, and a very frequent symptom of tuberculosis is an impaired digestion. Now, while I would not wish to say that bad teeth constitute the only cause of these digestive disturbances, if bad teeth are present, they are contributors to these pathologic conditions. Ulcerated teeth may give entrance into the bone to tubercle bacilli accidentally inhaled, or as the result of secondary infection.

Since it is impossible to control dental hygiene except among children, and, may I also say, equally impossible to teach the subject to the majority of adults, modern preventive medicine has very wisely directed its attention to the children, and of course to those attending the public schools. It has been stated that, if New York children are typical of the school children of the United States, there must be in the schools of this country 12,000,000 children having physical defects, more or less serious, that should receive attention from parents and family physicians. Of the school children in New York City and in the United States there must be handicapped by malnutrition, 41,000 in New York; 1,248,000 in the United States; enlarged glands, 5,460,000 in the United States; bad teeth, 8,988,000 in the United States; defective breathing, 7,092,000 in the United States. Dr. A. H. Merritt informs me that recent investigations have shown that no less than 95 per cent. of the children in a public school are suffering from decayed teeth.

It is not within my province to speak of the manifold medical, esthetic and even economic aspects of bad teeth in children or adults. I only desire to emphasize the great need of good, sound teeth in the prevention of tuberculosis on the one hand, and in the treatment of it on the other. The child or adult having good teeth will masticate his food properly and thus digest and assimilate it to better advantage. I have already said that we cannot reach the adult population, at least not nearly as easily as we can reach the children. It is for this reason that our local health department has very wisely created a division of child hygiene, with Dr. S. Josephine Baker at its head. The first thing to do, if one wishes to accomplish anything in the line of dental hygiene among children, is to instruct the parents; and to this end our New York Health Department issues the following leaflet and distributes copies freely to all parents through the children:

ISSUED BY ORDER OF THE BOARD OF HEALTH.

DEPARTMENT OF HEALTH
THE CITY OF NEW YORK.

### INSTRUCTIONS TO PARENTS REGARDING THE CARE OF THE MOUTH AND TEETH.

The physical examination of school children shows that in many instances the teeth are in a decayed and unhealthy condition.

Decayed teeth cause an unclean mouth. Toothache and disease of the gums may result.

Neglect of the first teeth is a frequent cause of decay of the second teeth.

If a child has decayed teeth, it cannot properly chew its food. Improperly chewed food and an unclean mouth cause bad digestion, and consequently poor general health.

If a child is not in good health, it cannot keep up with its studies in school. It is more likely to contract any contagious disease, and it has not the proper chance to grow into a robust, healthy adult.

**If the child's teeth are decayed, it should be taken to a dentist at once.**

The teeth should be brushed after each meal, using a tooth-brush and tooth-powder.

The following tooth-powder is recommended:

2 oz. powdered precipitated chalk.
½ oz. powdered castile soap.
1 dram powdered orris root.
Thoroughly mix.

This prescription can be filled by any druggist at a cost not exceeding 15 cents.

The child should take the tooth-brush and powder to the school and receive instructions from the nurse as to their proper use.

To give an idea of the scope of the work of this department, let me give a summary of its work as far as dental hygiene is concerned.

The Division of Child Hygiene of the Department of Health, through its medical inspectors and nurses detailed to duty in the medical inspection and examination of school children, gives each child in the public schools a complete physical examination. This includes an examination of the teeth.

During 1909, 231,081 children were examined of whom 131,747 were found to have defective teeth.

Of these latter, treatment was provided through the dispensaries and private dentists of the city for 4,616 children, of whom 2,591 had fillings and 2,025 had extractions. Each child was thoroughly instructed in the hygiene or the mouth. Children are assembled in the schools, in groups, by the nurses and are taught the use of the tooth-brush.

These instructions are repeated at the home each time that a nurse makes a home visit for any purpose whatever. At these home visits the instructions are made inclusive, so that they may be taken advantage of by all the children in the family and especially by those who have not yet reached the school age. A copy of the above quoted circular is given to each child in the school and left by the nurse at each home visit.

The circulars referred to are available for distribution by any agency in the city which requests the supply, and are at present in use in the schools of the Children's Aid Society and many of the other free schools of the city. This gives an idea of dental hygiene in its relation to tuberculosis as far as it can be accomplished among the pupils of our public schools.

The modern treatment of tuberculosis consists in the judicious use of fresh air, plenty of good food, plenty of good pure water inside and outside, careful regulation of rest and work and medical supervision. The proper feeding of the patient is one of the most essential factors. It cannot be done if the patient has bad teeth. It is very easy for us physicians to say to a patient when we examine him and find his teeth defective, "Have your teeth attended to." We say this daily to our poor consumptives, but I need not tell you why so many of them do not obey our instructions. There are very few free dental dispensaries in this city and the same may be said of many other cities; and many dispensaries require, and perhaps justly so, that the patient pay at least the cost of the material—amalgam, etc. We have in the greater city of New York alone from 40,000 to 50,000 consumptive poor. I do not believe that I exaggerate when I say that the majority of them need dental attention, and I know that it is a fact that only a very small minority get it. Some of our largest tuberculosis sanatoriums and hospitals in this city are without a dentist and all the dental work that is done is performed by the medical interns, and consists in extracting painful teeth. I do not believe that this is considered good dentistry in the light of modern dental science. Yet it is done every day. There are hundreds of the consumptive poor in our institutions who should have an entirely new set of teeth because they have but a very few bad teeth left; and still we say to them, "Chew your food well and eat plenty." How can we expect these patients to chew well and eat plenty of food and get well when they are thus handicapped in the most essential part of the treatment?

It was not my purpose in this paper merely to tell you of the relation of modern dental hygiene to the tuberculosis problem. I would be remiss in my mission did I not plead with you and particularly with men and women of wealth, to help us in the solution of this phase of the tuberculosis problem. One way to help is to create more dental dispensaries, and so to endow them that good work can be done, the workers paid, and the material furnished gratuitously to worthy patients. Every sanatorium or special hospital for consumptives should have one or several dentists attached to its staff according to the number of its patients, with a special well-equipped office, and an abundance of material needed for the efficacious treatment of all patients whose teeth need attention.

Here is certainly a field of true philanthopy and a splendid opportunity to serve a good and noble cause, and thus to be helpful in the attainment of that goal for which we are all striving—the eradication of the Great White Plague.—*Journal*, A.M.A., Aug. 13, '10.

